# Evolving Clinical–Translational Investigations of Cerebroprotection in Ischemic Stroke

**DOI:** 10.3390/jcm12216715

**Published:** 2023-10-24

**Authors:** Yinghui Li, Laurel E. Schappell, Claire Polizu, James DiPersio, Stella E. Tsirka, Marc W. Halterman, Neil A. Nadkarni

**Affiliations:** 1 Department of Neurology, Renaissance School of Medicine, Stony Brook University, Stony Brook, NY 11794-8651, USA; yinghui.li@stonybrookmedicine.edu (Y.L.); laurel.schappell@stonybrookmedicine.edu (L.E.S.); claire.polizu@stonybrookmedicine.edu (C.P.); james.dipersio@stonybrookmedicine.edu (J.D.); marc.halterman@stonybrookmedicine.edu (M.W.H.); 2 Department of Pharmacological Sciences, Renaissance School of Medicine, Stony Brook University, Stony Brook, NY 11794-8651, USA; styliani-anna.tsirka@stonybrook.edu

**Keywords:** ischemic stroke, cerebroprotection, translational studies, inflammation, stem cells, oxidative stress, excitotoxicity, ischemia-reperfusion injury

## Abstract

Ischemic stroke is a highly morbid disease, with over 50% of large vessel stroke (middle cerebral artery or internal carotid artery terminus occlusion) patients suffering disability despite maximal acute reperfusion therapy with thrombolysis and thrombectomy. The discovery of the ischemic penumbra in the 1980s laid the foundation for a salvageable territory in ischemic stroke. Since then, the concept of neuroprotection has been a focus of post-stroke care to (1) minimize the conversion from penumbra to core irreversible infarct, (2) limit secondary damage from ischemia-reperfusion injury, inflammation, and excitotoxicity and (3) to encourage tissue repair. However, despite multiple studies, the preclinical–clinical research enterprise has not yet created an agent that mitigates post-stroke outcomes beyond thrombolysis and mechanical clot retrieval. These translational gaps have not deterred the scientific community as agents are under continuous investigation. The NIH has recently promoted the concept of cerebroprotection to consider the whole brain post-stroke rather than just the neurons. This review will briefly outline the translational science of past, current, and emerging breakthroughs in cerebroprotection and use of these foundational ideas to develop a novel paradigm for optimizing stroke outcomes.

## 1. Introduction

Stroke is a highly morbid, heterogenous disease in which a focal neurologic deficit occurs due to ischemia or hemorrhage [1]. Ischemic stroke constitutes the majority of incident stroke cases, with 7.6 million incident cases and 77.19 million prevalent cases in 2019 worldwide [2]. Ischemic stroke occurs when a clot blocks an artery and impairs vascular perfusion and oxygenation of the brain. This process causes brain injury with lasting effects, including paralysis, language deficits, cognitive disorders, and even death. Current acute stroke therapies are predicated on promptly restoring perfusion by lysing and/or retrieving the clot by endovascular therapy (EVT), depending on eligibility criteria [3,4]. Despite reperfusion of carefully selected patients based on the predicted outcome, >50% of patients still do not achieve functional independence [4]. Expansion of the eligibility criteria (i.e., higher core volumes can still be eligible for EVT [5] or increasing window for thrombolytics [6]) will likely push the proportion of unfavorable outcomes to even higher numbers so long as there is some clinical benefit. This persistent poor outcome, despite reperfusion, is due to a combinatorial process of microvascular no-reflow [7] and ischemia/reperfusion injury (I/RI) [8]. Microvascular no-reflow is a condition with small-vessel flow failure despite macrovascular reperfusion [9]. I/RI, the result of restoring blood flow to ischemic tissue, places the potentially salvageable penumbra (an area of moderately reduced blood flow) at risk for irrevocable damage [10]. I/RI promotes the generation of toxic reactive oxidative species, increases the expression of cognate ligands on damaged endothelia that in turn promote inflammatory cell transendothelial migration (TEM) on damaged endothelia, and encourages a breakdown of the blood–brain barrier (BBB) [8,11,12,13,14,15]. Classically, drug development for stroke has focused on the concept of neuroprotection to limit secondary injury to neurons often mediated by I/RI. However, recent meetings by STAIR XI have sought to incorporate the entire neurovascular unit by switching from neuroprotection to cerebroprotection [16]. Cerebroprotection, also known as cerebral cytoprotection, would then address different components of neuroprotection (for neurons), glioprotection (for astrocytes), and vasculoprotection (for blood–brain barrier) to cover all aspects of the brain and brain recovery. Subsequently, the NIH has promoted the use of the phrase cerebroprotection in current requests for applications [17].

Notably, altering coagulation profiles, augmenting hemodynamic failure, and modulating elements in secondary cerebral damage to facilitate cerebroprotection have been extensively investigated and reviewed. To our group, clot lysis using thrombolytics and mechanical removal fall under indirect neuroprotectants and direct vasculoprotectants as their primary function is the “maintenance of circulatory patency or the reversal of vascular occlusion” [18]. These clot lysis drugs are the only available treatments for first time stroke patients [19], while most clinical stroke studies are focused on secondary prevention [20]. Developing drugs to promote cerebroprotection are thus paramount to improving stroke outcomes. Consequently, preclinical research serves to inform roles for escalation to clinical drug development. Unfortunately, several factors have been hypothesized to contribute to poor preclinical to clinical translatability including the young age of mice, shorter latency to receiving the experimental agent, genetic homogeneity of mice relative to humans, crucial differences in cerebrovascular anatomy and immunology, and outcome measures in preclinical vs. clinical studies [21,22]. This review will briefly address the preclinical–clinical translational failure using a reductivist approach by discussing the benefits and drawbacks of common therapies. We will then discuss the Stroke Preclinical Assessment Network and how it plans to work on issues in reproducibility in rigor. Ultimately, we will posit a novel paradigm for combinatorial therapies spread in time and space to guide future directions in approaching stroke with a precision therapy strategy. Notably, this review is not meant to be exhaustive nor fully comprehensive but is rather intended to lay the groundwork for understanding the path of the past as it guides our plans for the future.

## 2. Clot Lysis

Blood clots are currently purported to be aggregations of “fibrin, platelets, RBCs, white blood cells, VWF (von Willebrand Factor), and extracellular DNA” [23]. Their recanalization is expected to salvage brain tissue from injury. The first FDA-approved thrombolytic for stroke, the recombinant human tissue plasminogen activator (tPA), was approved in 1995 given its 28% reduction in 90-day disability as measured by the modified Rankin Scale (mRS) [3]. The original time constraints were for its administration to be given 3 h after the patient was last reported as normal [3], with extension to a 4.5 h window when excluding specific comorbidities (age > 80, high stroke scale, history of diabetes) [24]. As the literature describes, tPA solubilizes the clot and is catalyzed by fibrin to cleave plasminogen into plasmin [25], with eventual inactivation by circulating plasminogen activator inhibitor-1 (PAI-1) or neuronal neuroserpin [26]. The liver subsequently absorbs these inactive PAI-1/tPA dimers by scavenger receptor LDL Receptor Related Protein 1 (LRP1) [27]. As up to 6% of stroke patients treated with tPA develop hemorrhages due to blood–brain barrier breakdown, microvascular damage and non-thrombolytic actions of tPA, the very real risk for intracranial catastrophe makes tPA an imperfect drug [27]. Additionally, given its short half-life of 5 min (with a fibrinolytic activity of 7 min), tPA must be administered as an intravenous infusion with the risk of delayed hemorrhage. Despite its potency, even the original papers showed only 10% recanalization of proximal large vessel occlusions—the most severe ischemic stroke syndrome. Tenecteplase (TNK), a triple-mutant genetically modified alteplase, is purported to have pharmacokinetic advantages over tPA with reduced affinity for PAI-1, higher fibrin specificity, and increased half-life, allowing for single bolus administration rather than continuous infusion [28]. Early trials supported these claims with higher recanalization rates [29], but some later studies demonstrated non-inferiority [30,31]. A new study has just demonstrated that TNK may be 2.4-times more effective for early recanalization of thrombi longer than 10 mm in comparison to TPA [32], highlighting how physical clot characteristics may determine the drug choice. Due to time restrictions, only 3–8% of patients are eligible for intravenous clot lysis. Preclinical studies have focused on expanding the tPA time of the administration window by reducing the risk of hemorrhagic transformation. Studies on rodents mitigating BBB breakdown (reducing matrix metalloproteinase 9) and promoting vascular health (by boosting endothelial health) with the administration of drugs such as minocycline [33], or fasudil [34] could expand the tPA window of 4.5 h to longer time points with the consideration of combined adjusted tPA doses. Unfortunately, many of these studies have also shown conflicting success in preclinical and clinical trials to expand tPA windows. Although these drugs offer high recanalization rates, clots in the internal carotid artery terminus are described as three times larger than clots at the middle cerebral artery (MCA) bifurcation level and further resistant to lysis [35]. Drug administration alone was unlikely to address large vessel occlusions.

## 3. Mechanical Recanalization

Reduced efficacy for thrombolytics in large vessel occlusion led to the development of sophisticated mechanical retrieval devices and even direct clot aspiration as visualized in angiography. Original trials likely failed to show superiority due to suboptimal patient selection and the device’s need to retrieve the clot and leave the target vessel undamaged. However, the release of trials from 2015—REVASCAT [36], MR CLEAN [37], ESCAPE [37], SWIFT-PRIME [38], EXTEND-IA [39]—demonstrated that highly selected patients with an early presentation to hospital (<6–8 h) and minimal core burden could be eligible for mechanical thrombectomy and recanalization with a number needed to treat (NNT) of 2.6 (1 patient benefits for every 2.6 patients treated) to reduce disability on the mRS by at least one level [4]. Building upon these findings, DAWN [40] and DEFUSE-3 [41] sought to expand clinical criteria for inclusion in EVT. By accounting for variables such as age and real-time perfusion status as determined by perfusion-based imaging, these trials showed that expanding the time for EVT from 6 to 24 h still reduced disability despite an increased (but non-significant) risk for symptomatic hemorrhage compared to the controls. Further breakthroughs in EVT focus on recursive expansion of the inclusion criteria, showcasing a framework that suggests that testing novel therapies on highly selected patient populations with multiple modifications may pave the way for testing the limits of the therapy. For example, ANGEL-ASPECTS and SELECT-2 show that patients without favorable core/penumbra ratios (i.e., large cores) [42] can benefit from EVT [43]. MR CLEAN-LATE showed that patients with favorable collateral circulation status (without perfusion imaging) could still benefit from EVT [44]. Other recent studies have even tested the sequential order and route of administration of thrombolysis and EVT. The CHOICE trial, which was stopped early due to the COVID-19 pandemic, showed promising data that targeted intra-arterial clot lysis administration after EVT specifically targeted micro-vascular no-reflow with an adjusted risk difference of 18.4% for patients who received treatment compared to the placebo demonstrating a good outcome on the mRS [45]. However, trials investigating whether EVT could be pursued independent of tPA administration revealed mixed results [46,47,48,49]. Many clinical interventionalists expressed concern that bridging therapy (the administration of TPA prior to EVT) made clots more difficult to retrieve or resulted in distal clot propagation, effectively reducing cerebral perfusion despite large vessel recanalization. However, a 2023 retrospective study demonstrated intravenous thrombolysis, prior to ultimately unsuccessful EVT, offered better functional outcomes than EVT alone [50], given the inability to prospectively determine recanalization success in EVT. The gradual, iterative clinical practice of testing combinations and timing of thrombolysis and EVT has instructive lessons for developing future interventions.

## 4. Physiologic Manipulation

Since stroke is a failure of blood flow and oxygenation delivery to ischemic tissue, hemodynamic manipulation, oxygen supplementation, and temperature regulation have been explored in various studies. Flat head of bed positioning, despite early evidence showing improvement in the blood velocities of affected vessels without a concomitant increase in intracranial pressure [51,52], demonstrates no benefit in all stroke-comers with uncertain benefit per American Heart Association (AHA) guidelines [19]. Blood pressure management for stroke patients has been particularly difficult, as AHA guidelines do not account for reperfusion status or stroke volume [19]. Stroke has demonstrated U-shaped curves in relation to BP, in which both low BPs and high BPs have demonstrated poor clinical outcomes [53]. Blood pressure augmentation with phenylephrine has been studied in non-intervenable (aka TPA and EVT ineligible) noncardioembolic stroke with modest success in reducing early neurologic deterioration and improving outcomes [54]. Although there is some concern for inducing hemorrhages, a subset of patients for whom hemodynamic failure leads to worsening can be salvaged, given that cerebral autoregulation in the stroke brain is known to be impaired with documented evidence of worsened outcomes in patients with lower blood pressures [55]. As a corollary, despite notable practice pattern variation for BP goals post-EVT [56], targets for post-EVT BP goals are demonstrating trends that SBP > 158 will maintain good outcomes [57]. Evidence for oxygenation supplementation, whether at hyperbaric or normobaric pressures, has hit a roadblock due to futility [58,59] and imbalances of deaths despite biological plausibility. Consequently, the PROOF trial attempted to enroll patients to determine a role for penumbral protection before EVT but was ultimately terminated for futility [60]. Studies then progressed to study low-dose oxygen supplementation (rather than hyperbaric or normobaric pressure), with a pragmatic trial design showing no benefit in non-hypoxic stroke patients [61]. Lastly, temperature regulation by cooling—given its demonstrated protective effects in preclinical models for optimizing cerebral blood flow, anti-inflammatory effects, and modulation of cell death pathways [62,63]—shows no clinical benefit [64,65,66] with increased adverse effects of pneumonia, shivering, and need for airway control. Despite clear preclinical data favoring all these approaches with clearly demonstrable physiologic dysregulation, understanding which patients benefit as balanced against the risks of each therapy has proven to be elusive.

## 5. Oxidative Stress

The generation of reactive oxidative species (ROS) by the endothelium [67], microglia [68], astrocytes [69], and phagocytes [70] is a problem attributed to the act of ischemia-reperfusion. ROS are generally speaking an “umbrella term” with hydrogen peroxide H_2_O_2_ and superoxide O_2_^.−^ being the principal mediators for ROS-mediated damage, especially in the brain [71]. Other agents include hydroxyl radical (HO^.^), hypochlorous acid (HOCL), nitric oxide (NO) and peroxynitrite (ONOO^−^). These ROS are typically created intrinsically through damaged cells in the mitochondrial respiratory chain of microglia and astrocytes within the injured brain or extrinsically by enzymes from inflammatory cells that constitute the inflammatory response. NO, created by phagocytes, interacts with soluble guanyl cyclase to act as a vasodilator [72]. Given that the brain maintains high levels of polyunsaturated fats required to maintain its lipid membranes, the brain is particularly susceptible to oxidative species by lipid peroxidation [73]. ROS levels can “overwhelm antioxidant defenses: and trigger a series of pathophysiologic events including the inflammatory response, BBB disruption, apoptosis, and autophagy leading to neuron degeneration and apoptosis” [74]. Determining the levels of these molecules is extremely difficult given their transient nature. In fact, it has been well described that the dangers of ROS are mostly imputed through the benefit of purported antioxidants in preclinical models. Mechanistically, antioxidant-mediated cytoprotection works via multiple pathways: by inhibiting free radical production, scavenging free radicals, and increasing their degradation [75].

NXY-059, a water-soluble free-radical spin trap (an unsaturated diamagnetic compound that forms stable radicals by joining a free radical) was tested with the hope that it could mitigate against ROS damage [76,77]. Preclinical studies using rat models of permanent and transient focal ischemia showed reduced infarct size when NXY-059 was administered after four and five hours of ischemia onset. Primate studies using marmosets subjected to stroke also noted that NXY-059 decreases motor deficits and hemispatial neglect [78]. These experiments were particularly promising because the therapeutic dose in humans was well-tolerated without adverse effects [79]. SAINT I found a statistically significant improvement in disability by mRS in patients receiving NXY-059 within 6 h of stroke onset but not in neurologic functioning [80] as measured by the NIH Stroke Scale (NIHSS)—the best predictor of 3-month outcomes [81]. To further investigate this concept, another trial, SAINT-II, was performed with a larger sample size and unfortunately, could not confirm the results found significant in SAINT-I [82], with a pooled analysis of both trials also confirming no benefit [83]. The discrepancy between SAINT I and SAINT II brings into question the best way to capture functional ability after stroke. There were also concerns in these studies about statistical significance versus clinical significance and the inclusion of both tPA-treated and untreated subjects for the analysis of subgroup outcomes [84]. This key facet underscores the possibility that the very act of treatment itself may change the pathophysiology of stroke. A meta-analysis exploring the reasons for the failure of the SAINT trials highlighted inadequate test subjects as a primary likely reason for the mismatch results from the experiments performed on young, healthy, mostly male animals, which cannot necessarily be generalized to stroke patients who are often older and likely have comorbidities [85]. Instead of being truly brain penetrant, its mechanism of action was ultimately determined to be mostly ‘vasculoprotective’ given its presence on the abluminal side of the stroke [86]. Some more recent in vitro studies failed even to demonstrate efficacy in cortical slices [87]. Questions regarding the drug’s potency (independently and compared to other compounds such as vitamin E) were also raised [88]. Unfortunately, post-hoc analysis revealed the inclusion of some mice in preclinical treatment that did not demonstrate a stroke at all, attributed to insufficient ischemia more so than the success of the drug [84].

Though many other reactive oxidative species agents were studied, none continued to show benefit. Could the possibility of benefit in ROS be at play? This is less likely. A more plausible explanation is that ROS generation is so significant at such an early time-point. When there are only low levels of endogenous enzymes such as Cu/Zn superoxide dismutase, catalase, phospholipid hydroperoxide glutathione peroxidase (the principal components of the enzymatic antioxidant system [89]) within ischemic/reperfusion of the brain, it is possible that exogenous administration even minutes later may miss the narrow therapeutic window (more likely seconds) for intervention and is unable to catch up to the ROS burden. Good clinical data reveal decreased levels of endogenous antioxidants in patients with stroke [90]. Plausibly, the antioxidant effect on reactive nitrous species (RNS) may be detrimental, although there has been no evidence of the tested anti-oxidant species causing this effect. Nitric oxide (NO)—the most prominent RNS—has been demonstrated to have vasodilatory properties in stroke. Whether scavengers also exert collateral damage by depleting endogenous NO remains to be determined.

## 6. Excitotoxicity

Excitotoxicity has been investigated since the 1960s in various disease models [91,92]. Glutamate (Glut) is believed to be the principal mediator of excitotoxicity in stroke by binding to the N-methyl-D-aspartate (NMDA) receptor, thereby facilitating intracellular calcium (Ca^++^) release and triggering the pro-apoptotic cascade for neuronal death [93]. Since original studies established exhibited calcium overload as the primary mediator, further refinements in excitotoxicity have demonstrated that four critical aspects sequentially mediate excitotoxicity damage [93].

(1) Induction of NMDA-Receptor 2B (NR2B) receptors to raise [Ca^++^]i, thereby activating neuronal nitric oxide synthase (nNOS) and NADPH oxidase 2 (NOX2). This activation generates ROS and (RNS). These toxic byproducts are associated with Zinc (Zn^++^) overload following Ca^++^ influx;
(2) Amplification of the Ca^++^ influx signal by Ca^++^/Zn^++^ activation of other channels and feed-forward Glut release due to astrocytic swelling and ROS;
(3) Expression of multiple pathways mediating cell-death activated by Ca^++^/Zn^++^ overload, potassium (K^+^) efflux, hydrogen (H^+^) influx and oxidative stress to promote calpain-mediated death with severe necrosis in the core and enhancement of oxidative damage if reperfusion does occur;
(4) Promotion of inflammation and subsequent leukocyte infiltration.

Despite efforts to target glutamate-mediated excitotoxicity by employing glutamate competitive inhibitors, noncompetitive inhibitors, and modulation of Zn^++^, Mg^++^, and H^+^, various issues have led to translational failures. The first preclinical trials of drugs [94,95] showed failure clinically [18], likely due to drugs not being given at the stroke onset and thereby attempting to correct excitotoxicity when it is too late. There were also trends towards worse outcomes with some drugs depressing consciousness [93]. In fact, in response to the number of failed excitotoxicity drug trials, STAIR—the Stroke Therapy Academic Industry Roundtable—was created in 1999 in an attempt to improve the preclinical–clinical translation for all purported cerebroprotective agents [96].

To counter the timeliness issue, a clinical trial known as FAST-MAG attempted pre-hospital administration of Mg^++^—given that it is an NMDA antagonist by blocking the ion pore to limit Ca^++^ influx [97]. Unfortunately, despite being safe, no demonstrable difference in clinical outcome was noted. Within the excitotoxicity field, it is increasingly realized that tradeoffs between earlier and later pathways have benefits and harms. Understanding that interventions for excitotoxicity exist on a spatiotemporal continuum may guide future studies. Success will likely be dependent on modulating the proportion of necrosis (acute, swelling) to apoptosis (delayed, chromatin condensation) that occurs, as well as spatiotemporal effects of intracellular calcium modulation within the cell [93]. Given the complexity of excitotoxicity and its exquisite spatiotemporal constraints, interest in the field of stroke excitotoxicity has cooled compared to earlier decades. However, in 2022, genome-wide association studies [98] have confirmed that ADAM23 and GRIA1, two components of a neuronal excitability synaptic complex, may explain genetic variation in a small subset of patients with early neurologic deterioration post-stroke as measured by a change in the NIH Stroke Scale. Given the hesitancy to re-evaluate excitotoxicity with prior failures, this genetic study reinvigorates the need to continue exploring which patients are susceptible to excitotoxic damage and which ones are not.

## 7. Inflammation

Inflammation, the process by which white blood cells are recruited to damaged tissue, has long been sought for therapeutic intervention [99,100]. Beginning studies of the brain demonstrated clear leukocytic infiltration into the brain that began shortly after ischemia commenced [101]. Notably, the process of transendothelial migration in the brain has long been considered unique due to the brain’s unique “immune privilege” in healthy states [102], but even that concept has been challenged as of late [103]. Early trials built on the findings of leukocyte infiltration in both models of permanent ischemia and transient ischemia/reperfusion models [104,105,106]. Recently, a comparative paper between rodent models of stroke and human stroke found that leukocyte infiltration progressed predictably from neutrophils, monocytes/macrophages to T-cells over time, with a delayed peak of macrophages in human stroke compared to rodents [107] with some variation depending on the relation to the core/penumbra.

The contention between spatial location of entry of leukocytes and whether it matters has been difficult to navigate. Some preclinical work has shown that neutrophils enter the brain [108], others attest that neutrophils never cross the blood–brain barrier [109], and yet others state that neutrophils do enter but only after a certain amount of time [110]. Combined with the teleologic belief that there must be some purpose to inflammation (given how stereotyped it is), concerns have been voiced that the universal blockade of leukocyte entry may actually worsen outcomes by inhibiting stereotyped repair patterns or facilitating post-stroke immunosuppression. In addition to promoting direct cytokine-mediated inflammation, preclinical and clinical data show that leukocytes also can produce microvascular stalls [111] that impair microvascular re-flow and create thrombogenic neutrophil extracellular traps through crosstalk with platelets that promote inflammation and worse outcomes [112]. Critically, pial blood sampling of LVO patients undergoing EVT demonstrated that higher ischemic-to-systemic neutrophil ratios were associated with worse neurologic deficits in patients with stroke despite an equivalent reperfusion status [113]. The following subsections will describe some different strategies in the attempt to modulate inflammation.

### 7.1. Intercellular Adhesion Molecule (ICAM)-1 Blockade

Intercellular adhesion molecule-1 (ICAM-1) is a homodimeric ligand between leukocytes and endothelial vessels [114]. Blocking ICAM-1 interrupts the process of neutrophil adhesion to the endothelium and, in turn, prevented neutrophil transmigration out of the blood vessel in a rodent model of ischemia/reperfusion, but not in permanent ischemia [115]. Counteracting transendothelial migration decreases inflammation as leukocytes secrete inflammatory chemokines that may augment damage [116]. Testing an anti-ICAM antibody was partly inspired by preclinical evidence in animal models showing that anti-CD18 antibodies—which also disrupt leukocyte adhesion—decreased gastrointestinal injury after hemorrhagic shock, post-infarct myocardial injury, and neurologic damage after CNS ischemia [117]. In their study, Bowes et al. [117] used a rabbit cerebral embolism stroke model to show that rabbits receiving anti-ICAM 5 min after embolization require a larger clot to cause behavioral neurologic deficits at 24 h post-clot compared to control rabbits. Notably, rabbits receiving tPA 30 min after ischemia had similar outcomes to the anti-ICAM-treated group and rabbits with combination therapy of anti-ICAM plus tPA (30 min delay) did not have significantly different outcomes from the single-drug groups. No augmentation of the effect by anti-ICAM was similarly noted when the tPA delay was 90 min, although the outcome measure for tPA-only and combination therapy groups were both significantly different from the control groups. While the similar mean results for tPA-only and combination therapy groups make it difficult to highlight anti-ICAM’s contribution, the significant results in the anti-ICAM-only group might point to a cerebroprotective potential. It should also be noted that the variance in outcome measures is lowest in the combination therapy groups while it is larger in the tPA-only groups, further suggesting the cerebroprotective potential for anti-ICAM. Based on preclinical studies, enlimomab was hypothesized to reduce the infarct volume and improve post-stroke functional outcomes by reducing leukocyte adhesion. Unfortunately, the clinical results of the Enlimomab Acute Stroke Trial suggested no improvement in patients receiving enlimomab post-stroke and worse outcomes, including fever and pneumonia that manifested with increased dependency and even death [118]. These findings stoked fears of the adverse effect of modulating the immune response post-stroke [118]. A study investigating the failure of the enlimomab clinical trial found that rats administered murine anti-ICAM formed antibodies to the drug, which activated an inflammatory response and activated neutrophils [119]. This group hypothesizes that a similar immunologic response to belimumab—the concern that enlimomab may not have been fully humanized—in humans may have contributed to the worse clinical outcomes in patients receiving it in an inflammatory process like stroke [119]. The conduction of this study additionally highlights a tentatively fruitful method to evaluate the failure of cerebroprotective clinical trials and use identified problems and shortcomings to maximize the potential of future studies.

### 7.2. Very Late Antigen (VLA)-4 Blockade

Given the ambiguity between endothelial adhesion molecules, incomplete blockade of leukocyte extravasation had been identified as a significant limitation of targeting distinct adhesion molecules and may have partially contributed to the lack of clinical success observed with ICAM blocking strategies. Consequently, upstream integrins involved in leukocyte transendothelial migration have been posited as potentially attractive targets to induce a more substantial blockade of cell influx. One therapeutic strategy is natalizumab, an anti-VLA4 antibody that blocks VLA4-Vascular Cell Adhesion Molecule (VCAM)-1 interaction-mediated T-cell invasion [120]. VCAM-1 is an upstream endothelial ligand prominent in brain microvasculature. In addition to their direct contribution to pro-inflammatory cytokine signaling and induction of apoptotic pathways, T cells have also been demonstrated to modulate microglial polarization in the ischemic core and, thus, represent a potential means to target both infiltrating and resident immune cell activity following a stroke [121]. Preclinical studies investigating the effects of VLA4 blockade demonstrated decreased infarct volumes and improved behavioral outcomes following permanent MCAO and transient 30 min MCAO when anti-CD49d (targeting the alpha subunit of VLA4) was administered 24 h before occlusion or 3 h after surgery [122]. Blockade of VLA4 also decreased the infiltration of lymphocytes and granulocytes, as well as activation of resident microglia and monocytes, suggesting a widespread role for VLA4-mediated T cell influx in the post-stroke immune phenotype. Lastly, VLA-4 blockade decreased the upregulation of VCAM-1 upregulation, thereby reducing leukocyte recruitment. Notably, all T cell subpopulations, including Treg cells, were observed to be decreased following anti-CD49d treatment, which may have harmful consequences in longer-term studies of post-stroke repair. Despite the promising preclinical data, when VLA4 blockade was assessed clinically in the ACTION I trial via administration of natalizumab up to 9 h after stroke, there was no reduction in infarct size at 5 or 30 days post-stroke as measured using MRI. Functional outcome measures following natalizumab treatment revealed variable results with a trend towards functional improvement. However, natalizumab failed to elicit any significant NIHSS changes at any of the time points studied.

A second trial, ACTION II, was conducted with a plan to further investigate functional status as a primary outcome rather than infarct size using MRI [123]. This trial assessed drug administration across a broader range of time from the patients’ last known normal state. In this cohort, natalizumab was associated with a decreased frequency of excellent patient outcomes, as defined by mRS 0–1 (indicating mostly an independent status with no significant disability). As in the previous clinical trial, natalizumab failed to improve the observed rates of adverse, serious, and death events. The discrepancy between functional outcomes in both clinical trials may be attributed to differences in favorable outcomes among the placebo groups; however, aggregation of patient outcomes from both studies demonstrated worse functional effects with natalizumab treatment. The lack of success in clinical trials assessing the potential of VLA-4 blockade may be due to inappropriate intervention time, as natalizumab was administered well before the peak of lymphocyte influx (more on the order of days), or an imbalance between the beneficial effects of blocking pro-inflammatory lymphocytes and the consequences of losing regulatory T cell function.

### 7.3. C-C Chemokine Receptor 5 (CCR-5) Blockade

Maraviroc, a drug originally developed against the human immunodeficiency virus (HIV), is the latest example of a repurposed drug making headway in the stroke literature. As a C-C chemokine receptor 5 (CCR-5) inhibitor, this drug was found in a Pfizer drug screen to inhibit the fusion of the gp120 HIV envelope protein to CCR-5 [124]. By doing so, maraviroc inhibited the entry of HIV into immune cells. Given that CCR-5 is expressed widely on monocyte-derived macrophages and that blockade of the molecule did not affect cell signaling, maraviroc is highly selective, does not confer cytotoxicity, and proves to be an extremely precise drug. Investigations by Joy et al. [125] found that although CCR5 is also expressed in normal brain microglia, post-stroke it is expressed in cortical neurons and decreases in microglia/macrophages. Consequently, CCR-5 exhibits clear spatiotemporal differences even in relation to infarct. CCR5 knockdown using maraviroc improves motor recovery in a mouse cohort. Patients with a naturally occurring CCR-5 genetic loss of function mutations showed improved stroke recovery. These results make it plausible that CCR-5 inhibition via maraviroc may promote stroke recovery. Although the MAROS trial investigating maraviroc for post-stroke motor recovery has not posted updates since 2020, the basic science–translational approach of looking at other drugs that can be repurposed towards stroke therapy and recovery remains compelling.

### 7.4. Interleukin-1 (IL-1) Blockade

IL-1, a prominent pleiotropic cytokine released by the ischemic brain [121,122,123] has two prominent isoforms—IL-1α and IL1-β—that exert most of the molecule’s effects upon binding to the IL-1 receptor (IL-1R). Some of these effects include increased endothelial cell adhesion molecule expression and subsequent leukocyte transendothelial migration, increased blood–brain barrier permeability by matrix metalloprotein-9 release, promotion of astrocytic glial scarring, and toxic feed-forward release of other pro-inflammatory cytokines including interleukin-6 (IL-6) and tumor necrosis factor alpha (TNF-α). Preclinical studies showed that the administration of an IL-1R antagonist, an endogenous decoy receptor, can ameliorate IL-1 levels regardless of the route of administration. Consequently, recombinant human interleukin-1 receptor antagonist (rhIL1-ra) was created to dampen this chemokine release directly; it was thought to ameliorate long-term functional status by attenuating inflammatory markers correlated with poor outcomes in stroke patients [124]. Studies assessing the efficacy of rhIL1-ra indeed demonstrated a reduction in inflammatory markers, including IL-6, C reactive protein, and neutrophil count; however, there is conflicting evidence on whether this modulation improves clinical outcomes for patients. Critically, work by Salmeron et al. demonstrated that IL-1α blockade may be detrimental to inflammation, with the need for IL-1β to be specifically targeted [125]. Their work illustrates that IL-1α administration promoted vasculogenesis and neurogenesis with associated improved outcomes. Such findings raise the specter of whether the failure of the IL-1R antagonist may have been due to an incomplete understanding of cytokine biology—as blocking IL-1α led to conflicting findings in clinical trials. Such nuances reveal how difficult it is to develop and understand even one cytokine, let alone the many cytokines released throughout the spatiotemporal time course of stroke.

## 8. Stem Cell Therapy

To foster rehabilitation, stem cell therapy has emerged as a promising approach to elevate functional recovery following ischemic stroke. This potential mechanism includes establishing a novel neuronal network through transplanted stem cells and preserving the penumbra zone before it transitions into the core infarct. This protective effect is believed to be mediated by stem cell secretion of paracrine factors that promote anti-inflammatory and immunomodulatory effects, induce anti-apoptotic effects, and mobilization of endogenous neural stem cells.

Various types of stem cells have been used in clinical trials for ischemic stroke. Mesenchymal stem cells (MSCs) are widely used based on their self-renewable, differentiation, and secretory and immunomodulatory properties that promote the regeneration of damaged tissues and ease inflammation [126]. Bone marrow-derived mononuclear cells (BM-MNC) are relatively easier to obtain since autologous BM-MNC can be transplanted immediately after isolation from bone marrow [127], which is ideal for acute and sub-acute patients. Neural stem cells (NSCs) are optimal for replenishing lost neuronal networks. The currently used NSCs are commercial CTX-DP (conditionally immortalized neural stem-cell line). Endothelial progenitor cells (EPCs) are another stem cell derived from bone marrow that can differentiate into mature vascular endothelial cells. Still, their inherently low number in circulation limits their clinical application [126,127]. Some studies also test the effects of a mixture of two stem cell types. The doses of the transplanted cells ranged from 10^7^ to 10^9^, mainly within a single amount. Despite the wide range, no toxicity or severe adverse effects have been reported.

Stem cells are transplanted through different routes. Intravenous injection is the most accessible and widely used, but studies show only small quantities of cells can be found in the damaged brain tissue with IV injection, and most cells are trapped in the lungs [128]. Therefore, the secretion of neurotrophic factors resulting in the amelioration of apoptosis and inflammation are the main therapeutic mechanisms underlying IV infusion. The intra-arterial administration has exhibited superiority over IV injection by effectively transporting a larger cell quantity to the lesion site. Nevertheless, emerging findings reveal that many cells that migrate to the brain tend to vanish within 24 h after injection. This outcome signifies a failure to either survive or establish lasting engraftment [129]. Intracerebral administration directly injects the cells into the damaged brain tissues, which seems the most promising, but this approach necessitates surgical intervention, introducing the risk of secondary brain damage [126]. Most completed clinical trials were in phase I/II, testing the safety, feasibility, and dose effects.

We define acute phase infusion as SC transplantation within 14 days of stroke onset. Cells used at this time include autologous BMMNCs, which can easily be obtained from bone marrow [130,131,132,133], commercial stem cells such as MultiStem [133], and allogenic MSCs [132]. Cells were typically transplanted intravenously or intraarterially. The follow-up duration ranged from three months to two years, with all trials showing safety and feasibility, but most did not show significant efficacy. A study with a small number of patients (but with a control group) showed no difference in the mRS or NIHSS at 3 months between BMMNC transplantation and control groups. Still, an increase in plasma β-nerve growth factor levels was seen in the BMMNC group on day eight after transplantation [131]. The MASTERS (multipotent adult progenitor cells in acute ischemic stroke) study using intravenous transplanted MultiStem^®^ (invimestorcel) cells—which do not require immune suppression nor tissue matching—at doses of either 400 million cells, 1200 million cells or a placebo towards moderate non-lacunar stroke 24–48 h post ictus. showed no improvement in NIHSS after three months. However, according to their post-hoc analysis, patients who received cells between 24 and 36 h showed a significant improvement in motor recovery one year following treatment [131]. MASTERS-2 is currently underway to investigate this subset.

In the sub-acute phase (within six months of stroke onset), autologous MSCs and autologous BMMNCs have been widely used. Intravenous injection is still the most popular route, and intra-artery is also used through MCA or carotid. One study with a five-year extended follow-up period showed a decreased mRS score and an increased number of patients with an mRS of 0–3 in the MSC group [134]. Another study showed autologous MSC transplantation resulted in consistent improvement in BI and mRS compared with the control during the 3-, 6-, and 12-month follow-up periods [135]. However, other studies showed no effects on mRS, NIHSS, or BI with any cell type during follow-up periods of 3 months to 2 years [136,137,138,139]. Three studies showed significant improvement in motor function, including motor-NIHSS, motor Fugl-Meyer scores, and task-related fMRI activity [137,140] or lower extremity motor function [139], and neuroimaging showed interhemispheric connectivity and ipsilesional connectivity significantly increased in the MSC group [140], indicating stem cell-based therapy can enhance positive changes in network reorganization to facilitate motor recovery after a stroke.

Various cell types have been used during the chronic phase (over six months of stroke) due to the relative limitation on timing. Intra-cerebral infusion of cells is widely used in this phase. The post-transplantation impact through the IV or IA route primarily relies on their secretory and anti-inflammatory functions. However, during the chronic phase, inflammation is typically already established. Hence, the usage of IV or IA routes needs to be extensively employed. Transplantation via intra-cerebral injection has been demonstrated to be safe in all studies. Although randomized control studies are lacking, some studies show improved neuronal function following stem cell infusion compared with baseline, including ESS, NIHSS, Fugl-Meyer scores, F-M motor scale scores, BI, mBI Berg balance scale and Fugl-Meyer modified sensation [136,141]. Since there are no effective treatments for patients at this phase, large, randomized control studies urgently need to prove the efficacy.

### Putative Reasons for Stem Cell Failure

Based on the above outcomes of the completed trials, improvement of neuronal function, especially the motor function, has been seen in the trials for sub-acute and chronic phases. However, many studies showed no efficacy of stem cell treatment, which is different from the preclinical outcomes. The insights into the failures observed during the completed trials could unlock the pathway to the future success of stem cell treatments for ischemic stroke.

There are multiple reasons for the lack of success in stem cell therapeutics. First, low patient enrollment may be a primary reason that no efficacy was observed. Some studies enrolled no more than twenty patients. Even with an appropriate control group, reaching statistical significance for one or more clinical outcomes would be difficult. Second, in many trials, the criteria for stroke type were only “ischemic stroke” within the specific time window. This is understandable due to the difficulties of recruiting enough patients. However, more specific stroke types may lead to positive results. A difference in stem cell therapy effectiveness between white matter and gray matter predominant strokes seems likely. Third, the evaluation of neuronal function, including the parameters utilized, constitutes another crucial element that significantly influences the outcome. NIHSS and mRS are widely used. However, based on the current studies, motor function may benefit evidently from stem cell therapy. Therefore, more granular scales for motor recovery specifically may be necessary to demonstrate efficacy. Fourth, stem cell infusion timing may prove to be critical, especially for the acute phase. Therefore, the accurate windowing for stem cell infusion needs further investigation. Inherited limitations of the stem cells, such as the large size of certain types of stem cells, can lead to the trapping of stem cells in the lung via intravenous application and in the brain vessels via intra-arterial application, which can not only prevent the cells from reaching the brain but also cause secondary injury of the tissues. Although many preclinical studies show promising outcomes of stem cells in treating ischemic strokes, the clinical work was not as good. The discrepancy between preclinical and clinical studies could be attributed, at least in part, to the contrasting regenerative capabilities observed in typically young and healthy animals versus aged patients, often dealing with a range of chronic conditions. Studies show that the proliferation and angiogenic capacity of endothelial progenitor cells and MSCs were impaired in patients with coronary artery disease and metabolic disorders [142]. Additional investigations are needed to scrutinize the impact of stem cell therapies for stroke within the context of aged animals with chronic diseases. An incomplete understanding of comorbid factors and inclusion criteria for stem cell therapies may serve as a good area of focus for investigating future directions of this therapeutic modality.

## 9. Pleiotropic Drugs

Given the multitude of failures of targeted drugs, generally exploring the field of pharmacologic agents for post-stroke cerebroprotection has also been a recourse for investigation. Several clinical trials have aimed to generate cerebroprotective agents by targeting multiple mechanisms in the hopes of having various effects.

Nelonemdaz, a combination of aspirin and sulfasalazine (which is itself a conjugate of 5-aminosalicylic acid and sulfapyridine), was created in 2007 with the hopes that the novel drug can act synergistically better than either drug separately [143]. Aspirin is primarily administered to stroke patients for secondary prevention of stroke, as it promotes the blockade of platelet aggregation by irreversibly blocking cyclooxygenase and thereby limits conversion of arachidonic acid to thromboxane A_2_, limiting platelet aggregation [144]. However, it also demonstrates NMDA antagonism at supraphysiologic levels. Sulfasalazine demonstrates potent NMDA antagonism as well as ROS scavenger properties. Consequently, testing the combined drug preclinically for its anti-NMDA, anti-oxidant, and anti-thrombotic properties appeared to be a triple threat for multi-modality drug development with strong preclinical results on behavioral, histologic, and cell culture read-outs that showed synergism better than either aspirin or sulfasalazine in isolation [143]. Consequently, nelonemdaz progressed towards the phase II clinical trial SONIC, in which the drug was administered immediately before reperfusion and re-dosed over the next 5 days as patients were assigned to either placebos, low-dose, or high-dose drugs [145]. Though the primary outcome for ‘good outcome’ on the modified Rankin Scale (0–2) at 12 weeks was not achieved, a statistically insignificant ordinal shift trending towards improved outcomes was noted for both low-dose and high-dose nelonemdaz. A new study, RODIN, is now underway to specifically test for ordinal shifts in the modified Rankin Scale as the primary outcome [146]. Although the drug has yet to demonstrate clinical benefit, the goal of targeting multiple mechanisms is a promising foray into targeting stroke as a syndrome rather than focusing on a single druggable agent.

Nimodipine, a second-generation dihydropyridine calcium channel blocker, is most commonly used in subarachnoid hemorrhage to promote clinical neurological improvement per AHA guidelines [147]. It was believed to work by limiting calcium influx into vascular smooth muscle. However, the absence of angiographic improvement of vasospasm despite the improvement in clinical outcome imputed the existence of alternate mechanisms [148]. Consequently, nimodipine is believed to be affected by diffuse cerebral ischemia (a separate but related entity to vasospasm). The drug may exert its effects on a host of mechanisms [149]—fibrinolysis, mitigating neurotoxicity, and minimizing cortical spreading depression (the propagation of mass neuronal depolarization associated with paradoxical vasoconstriction due to brain injury in SAH). Given nimodipine’s effects on diffuse cerebral ischemia, trials investigating ischemic stroke were necessary. However, multiple trials—invoking mechanisms of increased blood flow by limiting vasoconstriction—demonstrated no benefit [150], as well as a higher incidence of harm at higher doses [151]. The most significant concerns were for regionalized hypotension induced by the drug outweighing the cerebroprotective effects [152] with exploratory findings of the study finding that lower diastolic blood pressures in the treated group were associated with unfavorable outcomes. Additional concerns included nimodipine’s limited CNS penetration, which further risks regional hypotension to obtain clinically relevant doses [149]. The development of chitosan-conjugated nimodipine, which would activate in specific pH environments, raises the possibility of localized drug effects without untoward systemic effects [153]. Though far from being considered a main player in improving ischemic stroke, its prominence as the guideline-recommended primary drug for subarachnoid hemorrhage suggests that the best outcomes for ischemic stroke may come from a drug with multiple effects.

Statins specifically work by competitive, reversible sterol pathway inhibition of 3-hydroxy-methylglutaryl coenzyme (HMG-CoA reductase), leading to a reduction in mevalonate [154]. Blocking this rate-limiting step of cholesterol synthesis leads to statins reducing serum low-density lipoprotein cholesterol (LDL-C) and apolipoprotein B. Clinically, the role for statin therapy in reducing LDL and atherogenesis in cardiovascular disease is incontrovertible [155,156,157]. Multiple studies have also confirmed statin benefits for stroke [158,159,160]. However, primarily due to the reduced production of isoprenoids further downstream, the role of pleiotropic statin effects in stroke patients has also been an area of investigation [161]. Isoprenoids are critical for the functioning of downstream GTPases including Rho. Rac and Ras [161]. Possible benefits of inhibiting these molecules include increased nitric oxide and subsequent endothelial function, reduced expression of inflammatory cell adhesion molecules such as ICAM-1, reduced ROS, reduction in pro-thrombogenic profiles, and improved angiogenesis [161]. Additional clinical data show some benefits for even non-atherogenic mechanisms of stroke such as cervical arterial dissection [162] and cardioembolic stroke [163]. Unfortunately, there is mechanistic difficulty in untangling the true pleiotropic nature of statins given that isoprenoid inhibition correlates with cholesterol inhibition and clinical trials for new statins would use current statins as a comparator arm [154]. Given that administration of statins is already standard of care for stroke secondary prevention [20], there is little controversy towards the utility of these drugs. Notably, patient compliance with statins, especially high-dose regimens, is critical for good outcomes [164]. Real-world data suggest ~10% noncompliance with these drugs due to the spectrum of myopathic disease [165]. In the future, isolating off-target statin effects in mechanistic terms may be crucial in understanding the differences in patients with statin compliance issues, especially as newer LDL reducing drugs may not exhibit identical pleiotropic effects that statins do [166].

## 10. Non-Pharmacologic Approaches

The role of non-pharmacologic approaches in promoting stroke recovery has also been an area of active investigation. Perhaps the most well-known modality is remote ischemic conditioning (RIC) for stroke patients. RIC utilizes repetitive inflation and deflation of a blood pressure cuff to elevated blood pressures above systolic measurement, with the hope of invoking a low dose I/RI. By doing so, this mechanism is hypothesized to promote an adaptive response to a larger I/RI (such as stroke). As described by others [167], RIC may promote (1) the release of autacoids (adenosine, bradykinin, calcitonin gene-related peptide) to activate afferent neural pathways or (2) the release of vasodilatory nitric oxide that can increase cerebral blood flow and protect against mitochondrial oxidative stress. In the initial studies, RIC was typically administered with four cycles of 5 min on and 5 min by an arm blood pressure cuff, ensuring that the cuff pressure is above systolic pressure for total arterial occlusion [168]. Two multi-center clinical trials investigating RIC have been conducted with conflicting results. The RESIST trial incorporated five cycles of cuff inflation/deflation on one upper extremity with prehospital initiation and continuation within the hospital setting, only to find no ordinal shift favoring functional outcomes in both ischemic and hemorrhagic stroke patients [169]. The RESIST trial benefitted from having a sham control cuff from which to compare results. In contrast, the RICAMIS trial was a randomized, open-label multi-center study that purposefully employed bilateral arm RIC with five cycles of 5 min on, 5 min off twice daily for 2 weeks over 10–14 days in addition to guideline treatment in comparison to standard treatment [170]. Patients were required to have acute moderate-sized ischemic stroke. This trial found that excellent outcomes (dichotomized into mRS 0–1 as excellent with scores 2–6 being poor outcome) were achieved for patients on a longer-term scale at 90 days without early neurologic improvement. This longer-term finding may suggest that RIC in this study supported recovery more so than cerebroprotection so to speak. These trials were the first multi-center investigations for RIC, with other pilot or single-center studies showing reduced tissue risk for infarct [171] or safety and feasibility with possible roles for improved neurologic outcomes [172]. The limitations of RIC include the difficulty in establishing a unified protocol for cycle duration and treatment duration, as well as the pleiotropic mechanism of action, which makes it difficult to isolate.

In brief, other types of non-invasive and invasive neuromodulation have been explored during both the acute and rehabilitation phases after stroke. The advantages of neuromodulation include the following: the stimulus is highly reversible, highly targeted, easily optimized, and very specifically timed. Non-invasive brain stimulation has been found to be effective in promoting recovery after stroke [173]. Cortical stimulation aims to restore the excitatory–inhibitory balance of the damaged brain and reorganize neural circuitry to enhance post-stroke recovery. Repetitive transcranial magnetic stimulation (rTMS) has been widely exploited to help the functional improvement of paretic limbs. High-frequency TMS increases cortical excitability and low-frequency stimulation decreases excitability [174]. Stimulation with transcranial direct current stimulation (tDCS) has been shown to be “probably effective” in improving motor function post-stroke according to a meta-analysis and systemic review [175]. Epidural electrical stimulation (EECS) as an invasive modulation offers greater stimulus with a more precise location. Preclinical studies and pilot human studies showed safety and improved recovery with EECS [176,177]. Vagus nerve stimulation (VNS) combined with rehabilitation has been shown to improve upper limb impairment after chronic ischemic stroke [178,179]. Acupuncture is recommended by the WHO as an alternative and complementary strategy for stroke recovery and has been demonstrated to improve balance, increase muscle strength, and reduce muscle spasticity [180]. Other emerging neuromodulation techniques include transcranial ultrasound neuromodulation (TUN) [181], optogenetics, and brain–computer interface [174] to promote neural plasticity. The possible mechanisms of neuromodulation in helping stroke recovery include restoring the excitatory–inhibitory balance, reorganizing neural circuitry, promoting survival signaling, promoting neurogenesis and cell proliferation, limiting apoptosis, and encouraging pyramidal tract plasticity [174,180,182]. Although current studies showed promising outcomes of neuromodulation, large clinical trials are needed to prove their safety and efficacy in stroke patients. Furthermore, a better understanding of the proper stimulation timing and sites will facilitate translation of this technique to clinical applications.

## 11. Bridging the Translational–Clinical Gap with SPAN

Due to the translational gaps between preclinical success and relative clinical failure, the NINDS, industry and leaders in the stroke field sought to develop a multi-arm, multi-site translational platform to encourage rigor, reproducibility, and quality control in efforts to develop a novel stroke drug [183]. By databasing behavior, employing longitudinal imaging, and utilizing a clinical trial approach, the project aims to facilitate high throughput and definitive answers on whether tested drugs in the late pre-clinical stage should progress to higher stages of investigation. By employing a CONSORT statement, interim analysis, and multiple sites, there were many aspects not before seen in preclinical studies. The original drugs tested were fasudil (a Rho-associated protein kinase inhibitor), fingolimod (a sphingosine 1-phosphate partial agonist limiting the release of monocytes from lymphoid tissue), uric acid (a peroxynitrite and hydroxyl scavenger), RIC (promotes cerebroprotection by the release of pro-vasodilatory properties), tocilizumab (anti-IL-6 receptor antibody), and veliparib (which inhibits the DNA repair enzyme poly (ADP-ribose)polymerase). All experiments utilized a 60 min transient middle cerebral artery occlusion model in rodents, with stage I involving young male and female mice, stage II with aged mice, stage III with mice models with comorbidities (aging, hypertension, diabetes, obesity), and spontaneous hypertensive rats and stage IV with young healthy rats. The regular inclusion of female mice was crucial given a lamentable lack of studying both sexes in preclinical literature. Additionally, accounting for the protective role of estrogen may be critical in understanding sex differences in stroke outcomes [184]. Behavioral testing was conducted with a baseline corner test (an assessment of motor asymmetry) 1 week before the stroke and and post-reperfusion at multiple time points. Intra-ischemic testing was pursued if the mouse was awakened during the ischemic period with a neurologic deficit score akin to a rodent stroke scale. Each stage underwent interim analysis using markers of futility or success and monitoring of MRI volumes to ensure consistent strokes. Notably, the primary outcome was the corner test—a motor assay in which mice enter a V-shaped entry and the symmetry of turns is recorded. Mice with strokes turn preferentially in one direction. With a primary behavioral outcome, SPAN clearly stakes its focus on clinical relevance given that most of the prior preclinical data presented studies’ lesion size as determined by histology and MRI. The corner test was specifically chosen because some behavioral tests show natural compensation over time [185]. Secondary outcome measures included the MRI-based lesion volume, etc. Limitations of the SPAN approach included no joint administration of tPA, a limited behavioral assay, and overall heterogeneity, which effectively recapitulates the reality of strokes in the clinical setting. It was announced at the International Stroke Conference 2023 through all the stages that uric acid had succeeded in the SPAN assessments throughout stages I–IV [186]. A second round of SPAN funding is now underway. Significant findings from the consortium demonstrate that heterogeneity is a part of the evaluation (whether the mouse is awakened during stroke, filament size, anesthesia duration, non-invasive monitoring of ischemia, and reperfusion) [187]. Although some may view that as a limitation, it is essential to consider that stroke itself is, in fact, highly heterogeneous, as demonstrated by multiple mechanisms for which cerebroprotection may need to be targeted for improving functional outcomes.

## 12. Discussion

**Figure 1:**
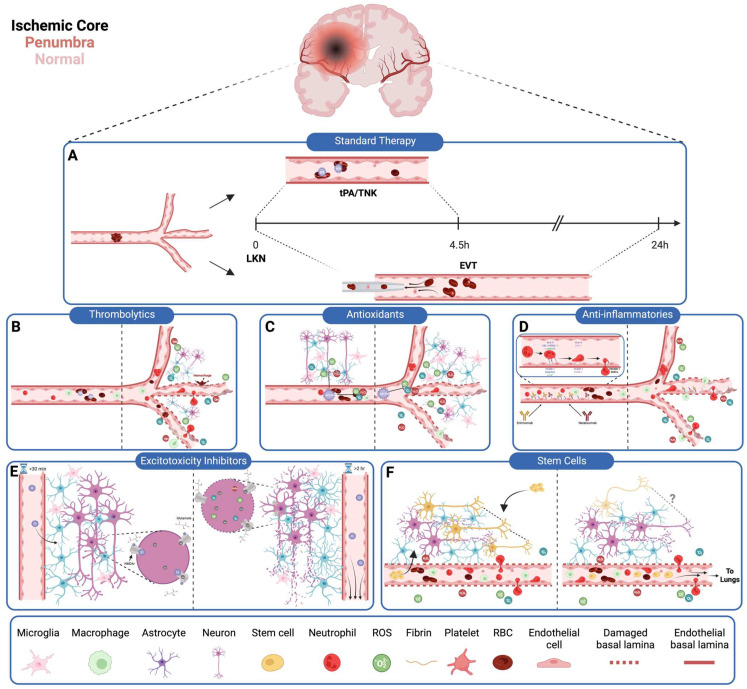
Current stroke therapies fail to comprehensively address the multifaceted mechanisms underlying ischemic injury and are often associated with unintended consequences or practical limitations. (**A**) Standard treatment involves thrombolytics (tPA/TNK) +/− endovascular thrombectomy (EVT). It depends upon the time since the patient’s last known normal status, comorbidities, a set of inclusion/exclusion criteria, and considerations of the volume of core versus penumbra. (**B**) Thrombolytics are intended to promote clot breakdown. However, their lytic action risks downstream clot propagation, endothelial barrier breakdown and subsequent hemorrhage, immune cell plugging, and expansion of the ischemic core due to neuronal effects of tPA. (**C**) ROS scavengers—NXY-059 in this example—would ideally trap ROS to render them inactive; however, due to excess of ROS creation in the injured brain and impaired penetrance past the blood–brain barrier, the amount of ROS substrate may be too difficult to overcome. (**D**) Antibodies such as enlimomab and natalizumab target signaling molecules involved in leukocyte recruitment, but redundancy in this pathway may allow for leukocyte extravasation via other adhesion molecules. Consequently, patients may be at risk for worsened post-stroke immunosuppression without affecting leukocyte transendothelial migration. (**E**) Excitotoxicity inhibitors aim to inhibit neuronal necrosis via antagonism of glutamate receptors. However, excitotoxicity-induced cell death begins within seconds of ischemia, so excitotoxicity inhibitors are delivered too late to mitigate damage. (**F**) Neural stem cells given intravenously or intrathecally are intended to replace damaged neurons. However, stem cell delivery to the infarcted tissue is suboptimal, with most cells being sequestered in the lungs.

**Figure 2:**
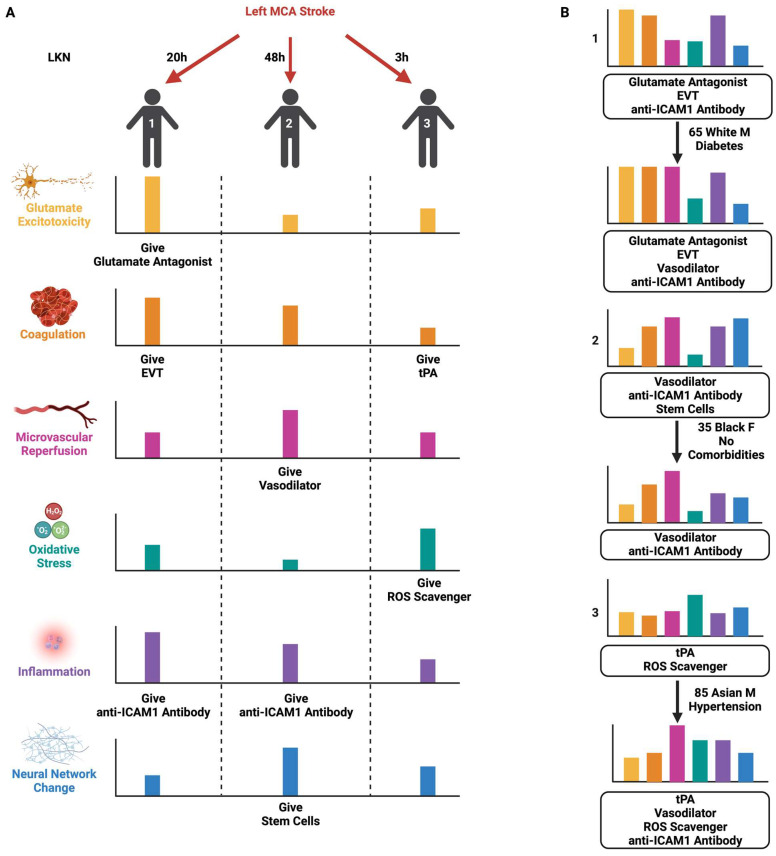
Emerging treatment strategies for stroke management should focus on a combinatorial approach as assessed by the patient’s individualized disease profile. (**A**) Patient 1displays a high level of coagulation and is, therefore, a candidate for mechanical thrombectomy given their time of presentation. Upon reperfusion, patient 1 demonstrates high glutamate excitotoxicity and inflammation levels, indicating that their individualized treatment plan should include an excitotoxicity inhibitor and an anti-inflammatory component. Patient 2 is not a candidate for either thrombolytic therapy or mechanical thrombectomy, so targeting glutamate excitotoxicity and oxidative stress are not viable options for this patient. Instead, patient 2 displays high levels of microvascular no-reflow, inflammation, and neural network change, suggesting a treatment strategy that may include vasoactive and anti-inflammatory therapies and stem cell administration. Patient 3 presents acutely and is a candidate for thrombolytics but unsuitable for mechanical thrombectomy. Following reperfusion, patient 3 demonstrates high levels of oxidative stress and should also be considered for antioxidant therapy. (**B**) For instance, patient 1 is at a higher risk for microvascular no-reflow given their pre-existing diabetes, which suggests a potential benefit for adding vasoactive therapy. Patient 2 is relatively young and has no comorbidities, and as a result, may be less likely to require intervention to repair neural networks. Patient 3 has pre-existing hypertension and is thus more likely to display microvascular no-reflow and inflammation, which may necessitate supplemental vasoactive and anti-inflammatory therapy.

One could easily surmise that the stroke field has false starts, contradictions, methodological concerns, and unforeseen challenges. However, this must be counterbalanced with high levels of promise and potential with shifting outcomes. In less than ten years, EVT transformed malignant stroke syndromes that invariably resulted in increased morbidity and mortality for conditions where patients can leave the hospital unscathed. With an NNT of 3, mechanical thrombectomy + thrombolytic is now one of the most effective treatments in modern medicine. Developing treatment regimens for those patients who do not benefit from EVT, as well as the high proportion of patients who do not have the option for EVT, is the focus of further cerebroprotective management. However, the road to future improvement will be complicated. Understanding the basic biology of stroke has proven difficult, as multiple mechanisms seem to be at play with a balance of benefits and untoward effects or limitations (Figure 1) Though many pathways can be implicated in contributing to poor stroke outcomes, it is apparent that blocking one aspect can either be overcome or rendered ineffective, depending on the condition. Consequently, a dynamic multi-drug cocktail to improve outcomes in stroke will likely need to account for (1) the timing of stroke, (2) patient physiology, and (3) patient stroke-related mechanisms of secondary injury. These factors would then need to be adjusted for patient attributes, including age, gender, race, and comorbidities (Figure 2). Such a spectral read-out would facilitate the best possible treatment at the earliest possible time point, with the ability to assess the effect of each intervention in real-time as well. Understanding which patients need further treatment and which do not would be helpful towards refining the failures of therapies listed in this review.

To achieve this aspirational goal, the stroke enterprise must not only focus on the development of new drugs but must also understand how to distinguish subsets of stroke patients that, as of 2023, currently seem to share a similar phenotype. This relies on the creation of more sensitive diagnostic and non-invasive tools. For example, although two patients with a significant stroke may seem to have similar deficits, the outcomes may be a sum of different factors for each patient. To complicate matters, there is clear preclinical evidence that spatiotemporal patterns constrain possibilities but also change throughout the hours, days, weeks, and months after stroke. This heterogeneity may seem daunting, but understanding these differences may lead to the development of more refined diagnostics and more precise treatments. Ultimately, we envision a successful therapeutic regimen involving continuous diagnostic assessments of the principal components and sum of each patient’s features, with recursive adjustments contingent upon real-time data.

## 13. Conclusions

Based on the outcomes of concluded clinical trials for therapies focusing on diverse stroke treatment modalities, further exploration through clinical and preclinical studies is imperative to unravel the intricate mechanisms and optimal interventions tailored to various stroke types and individual patient profiles. Given the time-critical nature of strokes, the dynamic nature of patients’ conditions fluctuates with timing. Therefore, developing and implementing therapies intricately attuned to the temporal context holds the potential to markedly enhance patients’ prognosis. Meanwhile, the combinatory therapeutic approaches hold promise in benefitting patients exhibiting diverse attributes. Like other diseases, stroke therapy necessitates a precision medicine framework considering the intricate interplay between patients’ genetic predispositions and environmental contexts.

## Data Availability

Not applicable.
